# Optical interventions to slow the progression of myopia

**Published:** 2019-05-13

**Authors:** Tim Fricke, Daniel Tilia, Minh Anh Tran, Helena Hurairah

**Affiliations:** 1 Senior Research Fellow and Paediatric Optometrist: Brien Holden Vision Institute, Sydney, Australia.; 2 Clinic Research Manager & Principal Research Optometrist: Brien Holden Vision Institute Limited, Sydney, Australia.; 3 Lecturer: Hanoi Medical University, and Optometrist, Vietnam National Institute of Ophthalmology, Hanoi, Vietnam.; 4 Consultant Ophthalmologist (Paediatrics and Strabismus): Brunei Eye Centre, RIPAS Hospital, Bandar Seri Begawan, Brunei, Darussalam.

**Bifocal spectacles, progressive addition spectacles, dual focus contact lenses and orthokeratology each appear to reduce myopia progression.**

**Figure:**
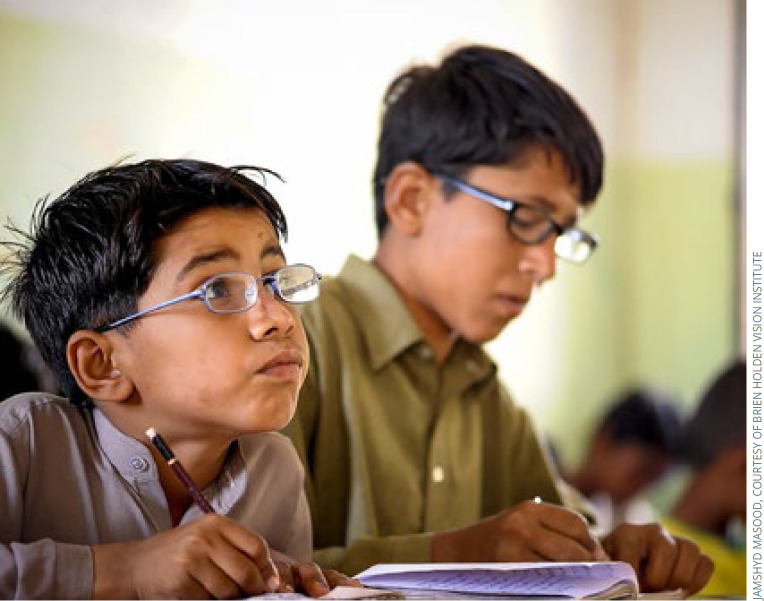
Spectacles improve academic performance and may assist in myopia control. PAKISTAN

People with myopia need vision correction (single vision glasses or contact lenses) to see objects that are far away. If their amount of myopia is increasing over time, there is a range of options to slow down the progression of myopia. This article will review the optical options that may be available where you work.

Myopia management is a challenge, because:

- We have an incomplete understanding of what causes myopia to develop and progress.
- Myopia usually starts early in life and people have to live with it for a long time.
- Myopia can progress over many years or even decades.

Eye care practitioners should stay up to date with the latest available evidence and involve patients and their families in deciding what treatment is best for them. Explain that current methods can slow down the progression of myopia, not stop or reverse it.

## Recommended interventions

### Progressive addition lens spectacles

Progressive addition lens (PAL, or multi-focal) spectacles provide distance correction and a near addition without a visible line in the lenses, meaning they can correct myopia and reduce accommodative strain in a cosmetically appealing form. Practitioners who thought accommodative strain was the main cause of myopia progression expected that PALs would reduce progression; however, research results have been disappointing.

PALs have been assessed in well-sized, randomised (between PALs and single vision spectacles), double masked, multi-centre trials with robust testing procedures.^1^ The studies used +2D near additions over each participant's distance refraction and monitored progression over 3 years.^1^ Findings included:

- Lower compliance and higher drop out in the PAL spectacles group compared to the single vision spectacles group, suggesting that some children did not like wearing the PALs.
- Across all participants, there was statistically significantly slower myopia progression in the PAL group in the first year, followed by equivalent progression between PAL and single vision spectacles.
- Across a subset of participants (children with high accommodative lag and near esophoria), there was an ongoing, cumulative, statistically significant reduction (24% on average) in myopia progression in the PAL spectacles group compared to single vision spectacles group.^1^

### Bifocal spectacles

Similar to PALs, bifocal spectacles provide a distance correction and a near addition, but with a visible line in the lenses, meaning bifocals are generally considered less cosmetically appealing. The near section of bifocal lenses can have different shapes (e.g., round- or flat-topped) and widths (e.g., 28 mm, 35 mm or executive/full width). If reduced accommodation strain is responsible for the small but significant reduction in myopia progression seen in specific children wearing PAL spectacles, results should be similar with any bifocal spectacles. However, myopia progression results may be different between PALs and various bifocal options if some other factor (e.g. dioptric demand across the visual field or peripheral focus) is more important in optically-mediated myopia control.

A randomised, controlled, but unmasked study compared myopia progression in Chinese Canadian children wearing executive bifocal spectacles compared to single vision spectacles. Myopic refractive error and axial length both progressed significantly slower in children wearing the executive bifocals: around 50% less than single vision spectacles. The reduced progression appeared ongoing and cumulative over 3 years, and were independent of binocular vision profile.^2^

On face value, improved myopia control in executive bifocals compared to PALs suggests that progression is determined more by dioptric demand across the visual field and/or peripheral focus, than by accommodative strain. However, design weaknesses in the bifocal study mean it would be useful to see the comparison replicated in a masked, multi-centre trial without ethnicity-specific selection.

### Dual-focus and multifocal soft contact lenses

Specifically designed soft contact lenses (e.g., dual-focus and centre-distance multifocal) provide a pattern of focus/defocus that animal models suggest will reduce myopia progression.^3^ The exact pattern of focus/defocus varies between lens types and manufacturers, but the centre of the lenses generally corrects the myopia permitting clear distance vision, while myopic blur is provided in the periphery with the intention of controlling myopia progression.

Randomised, masked studies have shown that both dual-focus and centre-distance multifocal contact lenses can reduce progression of both axial length growth and refractive change compared to single vision contact lens controls. Reductions of up to 80% in the first year have been published, but tend to average around 45% in the first year and a little less in subsequent years.^6^,^7^

### Orthokeratology

Orthokeratology (ortho-k) contact lenses are specifically designed to be worn while sleeping. They flatten the front surface of the corneas during the night, which reduces the eyes' optical power. The lenses are removed upon waking, but the corneas hold their new shape during the day, providing myopia correction without wearing any lenses during the day.

Ortho-k has been used for many years for myopia correction, but more recently was shown to affect myopia progression. While the exact mechanism for myopia control with ortho-k is unknown, peripheral myopic defocus appears to play a role. Modern ortho-K lens design creates a flatter central cornea and steeper peripheral cornea resulting in relative peripheral myopic defocus. Published studies have shown a reduction in the rate of increase of axial length of up to 50% over 2 years.^8^ Concern has been expressed regarding infection risk associated with ortho-K. While no significant adverse events were caused by orthoK in the largest longitudinal studies published,^8^,^9^ it is certainly worth giving due attention to maintenance of patient/lens hygiene and follow-up care.

**Figure:**
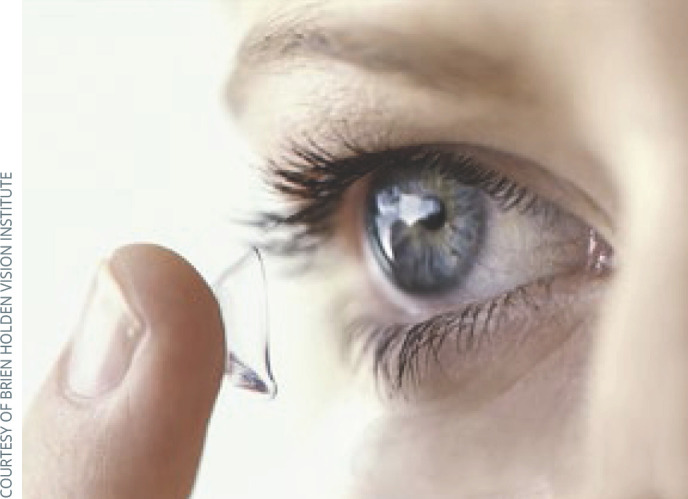
Orthokeratology lenses are worn at night to change the shape of the cornea. They have been shown to reduce the rate of axial elongation by 50%. AUSTRALIA

## Combination therapy

There are limited data available from randomised, controlled clinical trials as to what happens when interventions are combined; e.g., if low-dose atropine eye drops are combined with dual-focus contact lenses. However, a recent pilot study suggests that using orthoK with low-dose atropine is more effective in reducing the rate of myopia progression than ortho-k alone.^10^

## Not recommended

### Peripheral plus spectacles

Animal models have suggested that peripheral myopic defocus can produce central hyperopic growth that can override myopic growth signals from central vision.^3^ In practical terms, these spectacles could provide clear central vision while controlling myopia progression by blurring peripheral vision. However, the theories and models have not been translated to success in human trials. Peripheral plus spectacles are not a proven intervention and so we do not recommend them at this stage.

### Under-corrected spectacles

Evidence of the effect of under-correction of myopia is weak and mixed. There are some results that suggest faster progression with under-correction, while other results suggest slower progression with under-correction.^4^ We do not recommend employing this strategy unless more robust evidence suggests it is worthwhile.^5^

**Key messages**

Myopia correction should be carried out as early as possible to improve quality of life, productivity and educational performance.

Myopia, once present, progresses over a number of years. It is worthwhile considering the following optical interventions that reduce myopic progression as well as correct myopia:

- **PAL spectacles with +2D near addition** reduce progression by 24% over three years, but only for children with accommodative insufficiency and convergence excess.
- **Executive bifocal spectacles with +1.5D near addition** reduce progression by 50% over three years, but results need to be replicated in a multi-centre trial with masking.
- **Specially-designed contact lenses** (dual-focus soft, centre-distance multifocal soft, and ortho-k) can reduce myopia progression by up to 50% over 2 years. These tend to be expensive and are available mainly in high-income settings.
